# Oral-Systemic Interactions in Modern Healthcare: A Systematic Review of the Interrelationship Between Dental Pathologies and Systemic Diseases

**DOI:** 10.7759/cureus.104813

**Published:** 2026-03-07

**Authors:** Karthika Padmavathy, Ena Maakhni, Partha Sarathi Biswas, Firoz Sheikh, Tamanna Chhabra, Baijnath Das

**Affiliations:** 1 Department of Pathology, Sri Lalithambigai Medical College and Hospital, Dr M.G.R. Educational and Research Institute, Chennai, IND; 2 Department of Dentistry, All India Institute of Medical Sciences, Gorakhpur, Gorakhpur, IND; 3 Department of Public Health Dentistry, Kalinga Institute of Dental Sciences, Bhubaneswar, IND; 4 Department of Pathology, Raipur Institute of Medical Sciences, Raipur, IND; 5 Department of Prosthodontics and Crown and Bridge, K.M. Shah Dental College and Hospital, Sumandeep Vidyapeeth, Vadodara, IND; 6 Department of Medical Laboratory Technique, Teerthanker Mahaveer University College of Paramedical Sciences, Moradabad, IND

**Keywords:** autoimmune disorders, cardiovascular disease, oral microbiome, periodontitis, systemic inflammation

## Abstract

Periodontitis is a prevalent chronic inflammatory disease characterised by destruction of the supporting structures of the teeth, and increasing evidence indicates that periodontal inflammation and oral microbial dysbiosis contribute to systemic diseases through inflammatory, immune, and vascular mechanisms. This systematic review examined associations between periodontitis and cardiovascular, metabolic, autoimmune, neurological, and inflammatory outcomes in adult populations. Studies published from 2015 onward were identified using a structured literature search, including cohort studies, randomised controlled trials, case-control studies, cross-sectional analyses, Mendelian randomisation studies, and microbiome-based investigations. Due to heterogeneity in study designs and outcomes, qualitative synthesis was performed. Eleven studies met the inclusion criteria, demonstrating consistent associations between periodontitis and increased risk of hypertension, atrial fibrillation, rheumatoid arthritis, dementia, and elevated systemic inflammatory markers. Quantitative findings showed higher blood pressure, increased C-reactive protein levels, greater autoantibody positivity, enhanced inflammasome activity, and reduced oral microbiome diversity in individuals with periodontal disease. Periodontal therapy improved periodontal health and systemic inflammation, although cardiometabolic improvements were modest or inconsistent. The evidence supports periodontitis as a chronic inflammatory condition with significant systemic consequences, mediated by inflammatory, immune, and endothelial pathways, underscoring the importance of integrating periodontal health into disease prevention and management strategies in modern healthcare.

## Introduction and background

Periodontitis is a chronic inflammatory disease characterised by progressive destruction of the supporting structures of the teeth, including the gingiva, periodontal ligament, and alveolar bone [1]. In addition to the local forms of periodontitis, the disease is now known to have systemic implications [2]. The disease has a very high prevalence rate across the globe, and it is among the critical issues of public health as it has been linked to loss of teeth, poor oral performance, and quality of life [3]. Growing interest has focused on the investigation of how chronic periodontal inflammation can impact systemic physiology and give rise to or promote the progression of non-communicable diseases [4]. The mouth cavity is a special biological interface of exposure to external microbes and host immunological reactions [5]. The periodontal tissues are constantly subjected to multifaceted microbial communities, and the loss of homeostasis between the host and the microbe may result in chronic inflammation and dysbiosis [6]. Longitudinal dental and orthodontic interventions can alter oral microbial composition and periodontal parameters, which is relevant when considering oral-systemic pathways and immune priming mechanisms [7,8]. This inflammatory load does not necessarily stay in place of action, with inflammatory mediators, bacterial products, and immune cells able to enter the systemic circulation [9]. Periodontitis has therefore been suggested as a chronic inflammatory disease that has the potential to impact other distant organ systems along immune, vascular, and metabolic pathways [10].

There is growing epidemiological evidence that periodontitis is associated with a variety of systemic diseases such as cardiovascular disease, hypertension, metabolic diseases, autoimmune diseases, and neurodegenerative diseases [11]. Higher incidence rates of hypertension and atrial fibrillation have been shown by large population-based cohort studies in people with chronic periodontal disease [12]. Plausibility of a causal relationship between periodontal disease and high blood pressure has also been enhanced by genetic and Mendelian randomisation analyses, where there could be common cause inflammatory and endothelial processes [13]. Interventional studies on the outcomes of non-surgical periodontal therapy have demonstrated consistent progress among periodontal parameters and reductions in systemic markers of inflammation, including C-reactive protein [14]. Endothelial functionality and arterial hardness have also been reported to improve in some populations. But there has been a small or inconsistent change in blood pressure or glycemic control with periodontal treatment, especially in people whose hypertension or diabetes has been refractory [15]. These results indicate that the systemic impact of periodontitis could be facilitated mainly by chronic inflammation and not direct metabolic control [9].

Another significant field of study has been autoimmune and immune-mediated diseases. Longitudinal cohort research suggests that the number of patients who develop rheumatoid arthritis is much higher in people with periodontitis [11]. Immunological research has shown there to be an outcome between periodontal inflammation, oral microbial dysbiosis, and the synthesis of autoantibodies like anti-citrullinated protein antibodies [4]. Even in pre-clinical disease, patients with rheumatoid arthritis with high risk of the condition have been found to have altered oral microbiome profiles, diminished microbial diversity, as well as an enrichment of pathogenic taxa [15]. These results confirm the hypothesis of the mucosal origin of autoimmune diseases, where periodontal tissues can be the possible centres of immune priming. These epidemiological observations have been given a biological plausibility by mechanistic studies [5]. Periodontitis has always been linked to elevated levels of systemic inflammatory mediators, such as pro-inflammatory cytokines, inflammasome-related proteins, e.g., Nucleotide-binding domain leucine-rich repeat-containing protein 3 (NLRP3), and oxidative stress markers [16]. There is some evidence to indicate that periodontal inflammation could facilitate endothelial dysfunction, immune activation, microbial translocation, and dysregulated inflammasome signaling as well as support systemic disease processes [12]. Neurodegenerative outcomes that include Alzheimer's and vascular dementia further show that the chronic oral inflammation may have long-term effects on the entire system. Neurodegenerative outcomes that include Alzheimer's and vascular dementia have also been linked to chronic oral inflammation.

Although the association of periodontitis with systemic diseases is becoming increasingly evident, the strength, consistency and mechanism behind these associations are still under doubt. The current literature has diverse designs, populations, definitions of exposure, and outcome measures, which make it impossible to conclude on common grounds. The contribution of systemic inflammation, immune dysregulation, and oral microbial dysbiosis to each other is not fully comprehended. In addition, differences between epidemiological relationships and interventional results add to the questions of causality and clinical implications. There should be a synthesis of modern evidence, a thorough synthesis of which explains these relationships and determines common biological pathways between periodontal disease and systemic health outcomes. A synthesis of recent evidence is therefore required to clarify these associations and identify shared biological pathways between periodontal disease and systemic health outcomes.

The objective of this study is to systematically synthesise recent evidence examining the associations between periodontitis and systemic diseases, with particular focus on cardiovascular, metabolic, autoimmune, neurological, and inflammatory outcomes. The review evaluates epidemiological, interventional, and genetic evidence, and examines mechanisms involving systemic inflammation, immune activation, endothelial dysfunction, and oral microbiome alterations.

Accordingly, this systematic review was designed to address the following research question: in adult populations, is periodontitis associated with an increased risk of systemic disease outcomes compared with individuals without periodontitis? The primary outcomes assessed a priori were incident cardiovascular and cardiometabolic conditions (including hypertension), autoimmune outcomes (including rheumatoid arthritis), and neurological outcomes (including Alzheimer’s disease and vascular dementia). Secondary outcomes included inflammatory biomarkers and mechanistic evidence related to immune, endothelial, and microbiome-mediated pathways.

## Review

Methodology

Search Strategy and Search Terms

A methodological literature search was performed to find the studies that assess the relationship between periodontitis and systemic health outcomes, such as cardiovascular, autoimmune, metabolic, neurological, and inflammatory diseases. Some of the search terms were combinations of “periodontal disease”, “periodontitis”, “oral microbiome”, “dysbiosis”, “systemic inflammation”, “hypertension”, “rheumatoid arthritis”, “dementia”, “diabetes”, and “cardiovascular disease”. To reduce the risk of overlooking the specialty dental and orthodontic intervention studies that might be focused on the issue of the treatment-related oral microbiome perturbations and periodontal alterations, supplementary terms were added, such as the terms of “orthodontic”, “braces”, “aligners”, “periodontal therapy”, “scaling and root planning” and the term “dental intervention”, which was combined with terms related to the microbiome, including: “microbial diversity” and 16S rRNA [7,8].

Databases and Date Limits

Electronic databases such as PubMed, Scopus, Web of science and Google Scholar were searched. The only studies considered were those published since 2015 to make sure that they were up-to-date. The search was limited to studies published in the English language.

Study Selection Process

The selection of studies was according to Preferred Reporting Items for Systematic Reviews and Meta-Analyses (PRISMA) 2020. Relevance screening was carried out on titles and abstracts, and then a full-text assessment was carried out to determine eligibility. The PRISMA flow diagram [17] shows the selection process and the process of identification, screening, eligibility, and final inclusion process. The relevant reviews and reference lists of eligible studies were manually screened in order to identify other studies that were not identified by the main database search. Study screening and full-text eligibility assessment were conducted independently by two reviewers. Any disagreements regarding study inclusion were resolved through discussion and consensus. If consensus was not reached, a third reviewer was consulted to provide arbitration.

Eligibility Criteria

Studies had to be in an area that examined periodontitis or periodontal disease as the main exposure and had to involve an outcome of systemic health, such as hypertension, cardiovascular disease, autoimmune conditions, metabolic markers, neurodegenerative disorders, or systemic inflammation. Randomised controlled trials, cohort studies, case-control studies, cross-sectional studies, Mendelian randomisation analyses, and human microbiome studies were eligible as study designs. Exclusion criteria included studies where periodontal disease was not the main exposure, studies that did not analyse the systemic effects of an exposure, studies that were not conducted in humans or in vitro, and studies that did not provide enough methodological or outcome descriptions to allow meaningful interpretation.

Data Extraction

Full-text studies that qualified were carefully reviewed, and the main characteristics of the studies were then transferred to a standardised evidence table. The information that was extracted consisted of author and year, study design, population characteristics, periodontal exposure or intervention, systemic outcomes measured, and major findings.

Synthesis Approach

A quantitative meta-analysis was not performed because of the high clinical and methodological heterogeneity among the studies, such as differences in the definition of periodontal cases, systemic outcomes, and use of different follow-up periods and designs of the studies. The statistical pooling would have produced a large amount of heterogeneity (e.g., high I^2^ statistics), which would restrict interpretation. Based on this, a structured narrative synthesis was conducted to determine common patterns, mechanistic themes and convergent evidence in epidemiological, interventional, genetic and microbiome-based research.

Quality Assessment

Quality of methodology included the critical appraisal of the study design, clarity of research objectives, relevance of sample selection, validity of exposure and outcome measurement, control of confounding and transparency of reporting were used to evaluate quality of methodology. The longitudinal studies were examined to be sufficient in terms of the follow-up time and control of confounders. Clarity of the treatment protocols and outcome measures was evaluated in interventional studies. Microbiome and immunological studies were assessed according to the sampling methodology, analytical rigour and biological plausibility. On the whole, the included studies were rated as moderately to highly methodologically high.

Risk of Bias Assessment

Potential sources of selection bias, information bias, and confounding were assessed to determine the risk of bias. Observational studies were determined to be sufficient in matching or statistically controlling established confounders such as age, sex, smoking status and comorbidities. Interventional studies were analyzed on the possibility of performance and detection bias based on the delivery of treatments and outcome measures. The natural constraints of cross-sectional designs to provide temporality were admitted. Even though the possibility of residual confounding cannot be ruled out, the overall threat of bias of included studies was deemed to be satisfactory, as the results are similar across study designs and populations.

Results

Search Results

**Figure 1:**
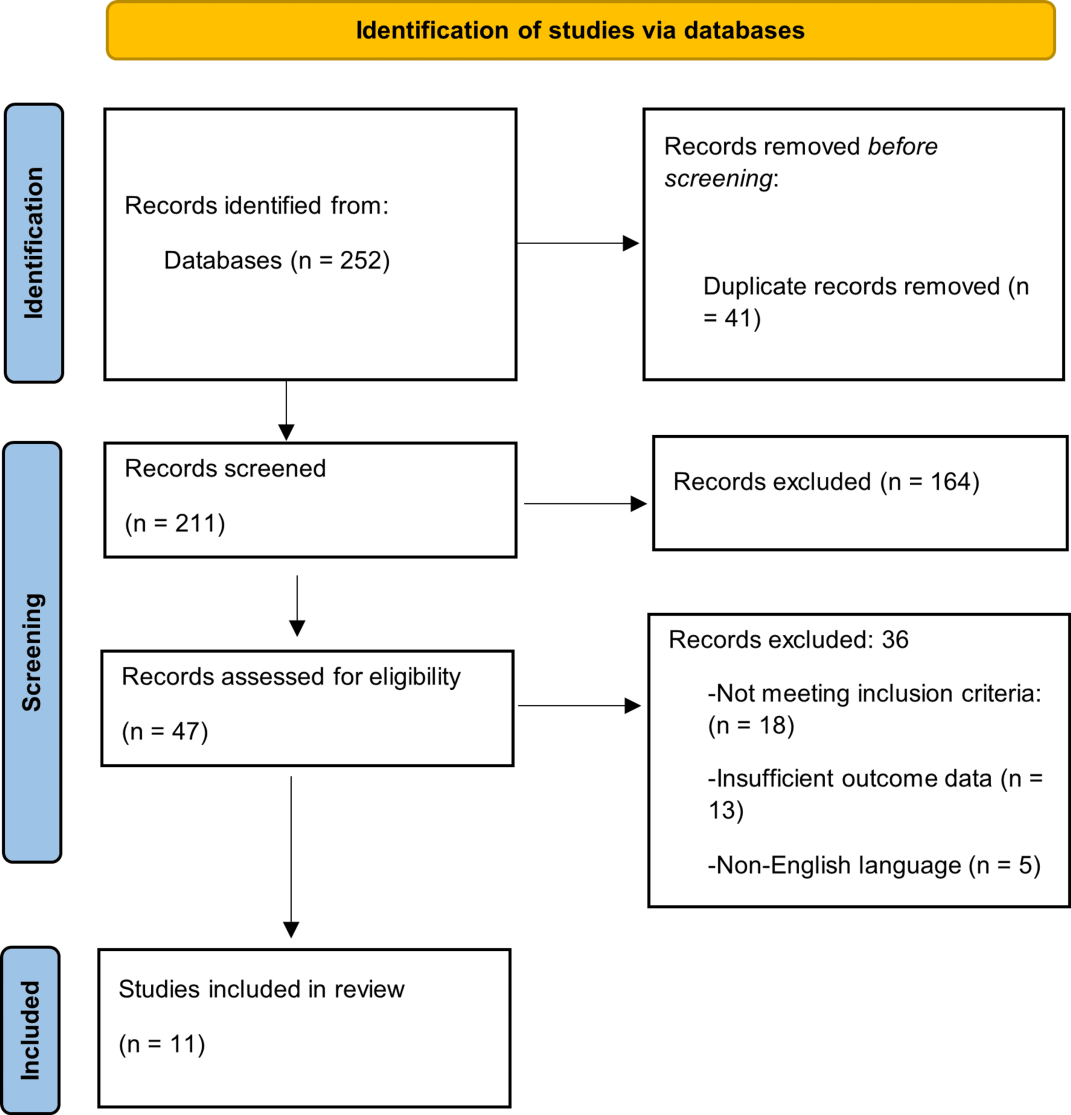
PRISMA flow diagram PRISMA: Preferred Reporting Items for Systematic Reviews and Meta-Analyses

A total of 252 records were obtained by the first search of the database. Upon the elimination of 41 duplicate studies, 211 records were left to undergo title and abstract screening. Among them, 164 records were excluded because they were not relevant to periodontal disease or systemic outcomes. The eligibility of the studies was evaluated on the basis of 47 studies in the full version. During this step, 36 studies were filtered out; the majority of the studies were not included due to either non-conformity to the pre-established inclusion criteria, lack of sufficient outcome data, or not in English. Finally, 11 studies were selected in accordance with all eligibility criteria and incorporated into the final qualitative synthesis. All the steps involved in the identification, screening, eligibility, and inclusion of a study are depicted in the PRISMA 2020 flow diagram (Figure 1).

Study Characteristics

Recent studies published between 2015 and 2025 were included and encompassed a variety of study designs, including nationwide cohort studies, matched cohort studies, randomised controlled trials, Mendelian randomisation analysis, case-control studies, cross-sectional research, and microbiome-based studies. Study populations were diverse and comprised the general adult population, patients with hypertension, diabetes, or rheumatoid arthritis, and at-risk populations like high-risk individuals with autoimmune disease. Various clinical periodontal parameters, diagnostic codes, tooth loss or oral microbiome profiling were used to measure periodontal disease. Systemic effects were hypertension and atrial fibrillation, dementia, rheumatoid arthritis, metabolism and inflammatory biomarkers, and indicators of immune dysregulation. Although the methodological diversity was noted, all the included studies evaluated periodontitis as the primary exposure variable and examined its association with subsequent systemic health outcomes. Key characteristics and major findings of studies that investigated the relationship between periodontitis and systemic health outcomes are discussed in Table 1.

**Table 1:** Studies linking periodontitis to systemic diseases AD: Alzheimer’s disease; AF: Atrial fibrillation; ACPA: Anti-citrullinated protein antibodies; BP: Blood pressure; CRP: C-reactive protein; HTN: Hypertension; IL-1β: Interleukin-1 beta; NSPT: Non-surgical periodontal therapy; PD: Periodontal disease / Periodontitis; QOL: Quality of life; RA: Rheumatoid arthritis; RCT: Randomised controlled trial; T2DM: Type 2 diabetes mellitus; VaD: Vascular dementia; 16S rRNA:16S ribosomal ribonucleic acid; NLRP3: Nucleotide-binding domain leucine-rich repeat-containing protein 3.

Study	Study Design & Population	Periodontal status/assessment	Intervention (if applicable)	Primary outcomes	Key findings / Conclusions
Kim et al. [18]	Nationwide retrospective cohort (Korea); n≈20,230; long follow-up	Severe periodontitis with tooth loss	Not applicable	Alzheimer’s, vascular & mixed dementia	Severe periodontitis with tooth loss is associated with increased risk of AD, VaD, and mixed dementia, suggesting periodontitis/tooth loss as a modifiable dementia risk factor.
Park et al. [19]	Nationwide cohort; n≈1.25 million	Periodontal disease status change (free/recovered/developed/chronic)	Not applicable	Incident atrial fibrillation	Chronic PD had the highest AF risk; recovery reduced risk vs chronic PD; new PD increased risk vs PD-free.
Czesnikiewicz-Guzik et al. [20]	Mendelian randomization (~750k) + RCT (n=101 hypertensive)	Genetic liability to periodontitis	Intensive non-surgical periodontal therapy	BP + endothelial function	Genetic evidence supports a causal PD → hypertension link; intensive periodontal therapy improved BP and vascular function.
Sherina et al. [21]	Case-control + immunology; periodontitis patients with ACPA-positive RA (n=3) and periodontitis patients without RA (n=4)	Gingival tissue inflammation and immune response to citrullinated periodontal antigens	Not applicable	ACPA/autoimmunity in RA	Supports the periodontal/mucosal origin of RA autoimmunity via cross-reactive responses.
Choi & Lee [22]	Nationwide matched cohort (Korea); n≈691,506	Incident periodontitis	Not applicable	Incidence of RA	Periodontitis is associated with higher RA risk (stronger in some subgroups); supports PD as a RA risk factor.
Mizuno et al. [23]	RCT; adults with type 2 diabetes mellitus and chronic periodontitis; n=40 (treatment n=20, control n=17)	Chronic periodontitis in patients with type 2 diabetes	Non-surgical periodontal therapy versus oral hygiene instruction alone	HbA1c, oxidative stress, QOL	NSPT improved oxidative stress balance and QOL; HbA1c improvement was not consistent overall (more apparent in poorer baseline control).
Rodrigues et al. [24]	Prospective intervention; refractory HTN + periodontitis (n=22)	Moderate-to-severe periodontitis	Non-surgical periodontal therapy	BP, CRP, periodontal indices	Periodontal parameters + CRP improved; BP reduction not significant, suggesting inflammation benefit without clear BP change in this sample.
Park et al. [25]	Nationwide cohort (Korea); n≈706,584; median follow-up 14.3y	PD status dynamics (free/recovered/developed/chronic)	Not applicable	Incident hypertension	Chronic PD had the highest hypertension risk; recovery associated with lower risk vs chronic; developing PD increased risk vs PD-free.
Hashimoto et al. [26]	Case-control; 85 RA vs 119 controls	Periodontal pocket depth and oral bacterial DNA	Not applicable	Odds of RA	Deep pockets and higher abundance of specific taxa are associated with higher RA odds; highlights the microbiome–periodontitis–RA link.
Bolyarova et al. [27]	Cross-sectional; n=108 (healthy / PD / HTN / both)	Periodontal disease with or without hypertension	Not applicable	NLRP3, IL-1β; metabolic markers	PD associated with higher serum/salivary NLRP3; serum NLRP3 showed predictive value for hypertension.
Tong et al. [28]	Cross-sectional microbiome study; 79 saliva samples (ACPA+ at-risk, RA, healthy controls)	Salivary microbiome composition (16S rRNA sequencing)	Not applicable	Oral microbiome diversity/composition; associations with ACPA/RF	Reduced microbial diversity and specific taxa shifts precede clinical RA and correlate with autoantibody levels.

Association Between Periodontitis and Cardiovascular and Metabolic Outcomes

Large population-based cohort studies demonstrated that chronic periodontitis was associated with an increased risk of hypertension and atrial fibrillation, while recovery from periodontal disease was associated with reduced cardiovascular risk [29]. Genetic analyses and interventional evidence further supported a potential causal relationship between periodontitis and elevated blood pressure, with periodontal therapy improving endothelial function and reducing systemic inflammatory markers in certain populations [30]. However, interventional trials involving patients with refractory hypertension or diabetes showed that although periodontal therapy improved periodontal status and reduced systemic inflammation, changes in blood pressure or glycaemic parameters were small or inconsistent [19]. Orthodontic and periodontal intervention studies in patients with preexisting periodontal conditions also demonstrated measurable improvements in periodontal parameters following treatment [10]. The findings suggest that periodontitis contributes to cardiometabolic risk primarily through chronic inflammatory mechanisms rather than through direct metabolic regulation. Table 2 summarises the cardiovascular and metabolic parameters assessed in the included studies.

**Table 2:** Cardiometabolic parameters assessed in the studies BP: Blood pressure; HbA1c: Glycated haemoglobin; mmHg: Millimetres of mercury

Study	Blood pressure parameters	Cardiac outcomes	Glycemic parameters	Systemic inflammatory markers
Park et al. [19]	Blood pressure diagnostic codes	Atrial fibrillation incidence	Not assessed	Not assessed
Czesnikiewicz-Guzik et al. [20]	Systolic and diastolic blood pressure; 24-h BP	Not assessed	Not assessed	C-reactive protein
Mizuno et al. [23]	Not assessed	Not assessed	HbA1c (%)	Oxidative stress markers
Rodrigues et al. [24]	Systolic BP, diastolic BP, mean BP	Not assessed	Fasting glucose, HbA1c	C-reactive protein
Park et al. [25]	Systolic ≥140 mmHg; diastolic ≥90 mmHg	Not assessed	Not assessed	Not assessed

**Figure 2:**
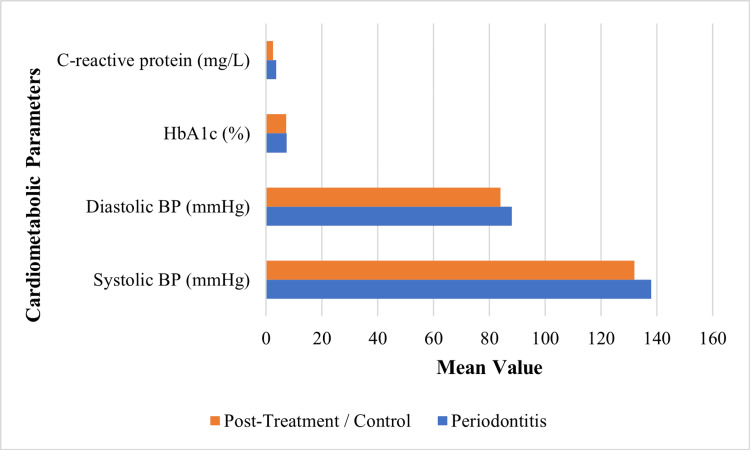
Cardiometabolic parameters in patients with periodontitis vs control BP: Blood pressure; HbA1c: Glycated haemoglobin; mmHg: Millimetres of mercury. Data derived from [20,23,24].

Figure 2 shows higher blood pressure, HbA1c, and C-reactive protein levels in individuals with periodontitis compared with post-treatment or control groups.

Periodontitis and Autoimmune and Immune-Mediated Diseases

Longitudinal cohort data revealed that individuals with periodontitis were at a higher risk of developing rheumatoid arthritis than periodontally healthy controls [31]. Immunological and microbiome-based studies further indicated that periodontal inflammation and oral microbial dysbiosis were associated with increased autoantibody production, including anti-citrullinated protein antibodies [32]. Changes in oral microbiome composition and reduced microbial diversity were also observed in individuals at high risk of rheumatoid arthritis prior to clinical disease onset [26]. These findings support the hypothesis that periodontitis and oral microbial dysbiosis may contribute to the initiation or progression of systemic autoimmunity. Table 3 summarises the periodontal, autoimmune, and oral microbiome-related variables assessed in immune-mediated studies.

**Table 3:** Autoimmune and oral microbiome parameters assessed in the studies ACPA: Anti-citrullinated protein antibodies; RF: Rheumatoid factor; 16S rRNA: 16S ribosomal ribonucleic acid; DNA: Deoxyribonucleic acid.

Study	Periodontal clinical measures	Oral microbiome assessment	Autoantibody measures	Immune parameters
Sherina et al. [21]	Probing depth, clinical attachment loss	Porphyromonas gingivalis antigens	Anti-citrullinated protein antibodies	Gingival B-cell activity
Choi & Lee [22]	Periodontitis diagnosis, tooth loss	Not assessed	Rheumatoid arthritis diagnosis	Not assessed
Hashimoto et al. [26]	Probing depth, bleeding on probing	Bacterial DNA quantification	Rheumatoid arthritis status	Not assessed
Tong et al. [28]	Not assessed	Salivary microbiome (16S rRNA sequencing)	ACPA and RF titers	Not assessed

**Figure 3:**
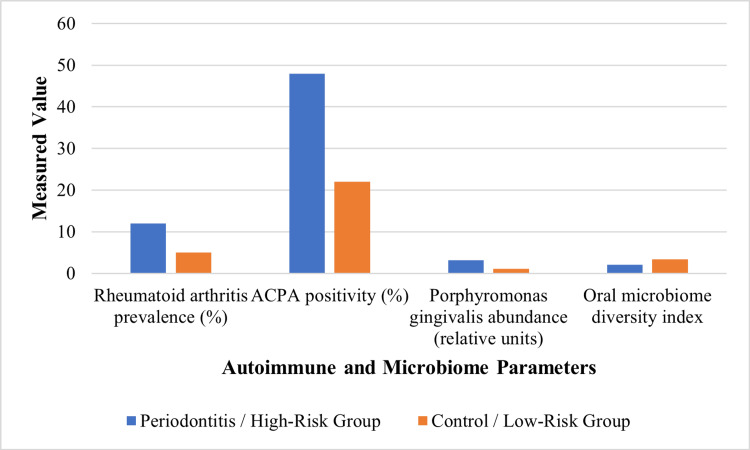
Autoimmune and oral microbiome markers in periodontitis ACPA: Anti-citrullinated protein antibodies. Data derived from [21,26,28].

Figure 3 shows higher autoimmune markers and pathogenic bacterial abundance, with reduced oral microbiome diversity, in patients with periodontitis compared with controls.

Systemic Inflammation and Biological Mechanisms Linking Periodontitis to Disease

In several studies, periodontitis was consistently associated with elevated systemic inflammatory markers, including C-reactive protein, inflammasome-related proteins, and pro-inflammatory cytokines [33]. Biomarker-based research further showed that periodontal disease was linked to increased concentrations of inflammatory mediators in both serum and saliva, and some markers demonstrated predictive value for systemic conditions such as hypertension [34]. Genetic, immunological, and microbiome-based evidence indicated that periodontal inflammation may influence systemic health through endothelial dysfunction, immune activation, microbial translocation, and inflammasome signalling pathways [21]. These findings support the biological plausibility of the observed epidemiological associations and reinforce the concept of periodontitis as a chronic inflammatory condition with systemic effects. Table 4 summarises the systemic inflammatory, vascular, and mechanistic parameters assessed across the included studies.

**Table 4:** Systemic inflammatory and mechanistic parameters NLRP3: Nucleotide-binding domain leucine-rich repeat-containing protein 3; IL-1β: Interleukin-1 beta; CRP: C-reactive protein.

Study	Inflammatory biomarkers	Vascular / Endothelial measures	Neurological or immune outcomes
Kim et al. [18]	Not assessed	Not assessed	Alzheimer’s dementia, vascular dementia
Czesnikiewicz-Guzik et al. [20]	C-reactive protein	Endothelial function, arterial stiffness	Hypertension
Sherina et al. [21]	Pro-inflammatory cytokines	Not assessed	Autoimmune activation
Rodrigues et al. [24]	C-reactive protein	Not assessed	Hypertension
Bolyarova et al. [27]	NLRP3, IL-1β, C-reactive protein	Not assessed	Hypertension status

Risk of Bias Assessment

Randomised controlled trials were evaluated using the Cochrane Risk of Bias 2 (RoB 2) tool, which examines bias arising from the randomisation process, deviations from intended interventions, missing outcome data, outcome measurement, and selective reporting [35]. Cohort and case-control studies were assessed using the Newcastle-Ottawa Scale [36], while cross-sectional studies were evaluated using the Appraisal tool for Cross-Sectional Studies (AXIS) tool [37], focusing on participant selection, group comparability, outcome assessment, and reporting quality. Based on domain-level judgements within each tool, studies were classified as having low, moderate, or moderate-high risk of bias. The detailed risk of bias assessments are summarised in Table 5.

**Table 5:** Study-level risk of bias evaluation of the included evidence RoB 2: Risk of bias 2; AXIS: Appraisal tool for Cross-Sectional Studies.

Study (citation)	Study design	Risk of bias tool used	Overall risk of bias	Key considerations
Kim et al. [18]	Retrospective cohort	Newcastle-Ottawa Scale	Moderate	Registry-based exposure assessment; residual confounding possible.
Park et al. [19]	Nationwide cohort	Newcastle-Ottawa Scale	Moderate	Periodontal status based on administrative data; confounding adjusted but not eliminated.
Czesnikiewicz-Guzik et al. [20]	RCT component	RoB 2	Low-moderate	Limited blinding typical for periodontal interventions; objective outcomes.
Sherina et al. [21]	Case-control	Newcastle-Ottawa Scale	Moderate	Small sample size; potential selection bias; exposure well characterized.
Choi & Lee [22]	Matched cohort	Newcastle-Ottawa Scale	Moderate	Matching reduced confounding; observational design limits causal inference.
Mizuno et al. [23]	Randomized controlled trial	RoB 2	Low-moderate	Small sample size; blinding not fully feasible.
Rodrigues et al. [24]	Prospective interventional study	Newcastle-Ottawa Scale	Moderate	No randomized control group; limited sample size.
Park et al. [25]	Nationwide cohort	Newcastle-Ottawa Scale	Moderate	Long follow-up; potential residual confounding.
Hashimoto et al. [26]	Case-control	Newcastle-Ottawa Scale	Moderate	Hospital-based recruitment; confounder control limited.
Bolyarova et al. [27]	Cross-sectional	AXIS tool	Moderate-high	Cross-sectional design; confounding and temporality limitations.
Tong et al. [28]	Cross-sectional microbiome study	AXIS tool	Moderate-high	Cross-sectional design; microbiome influenced by unmeasured factors.

Discussion

This systematic review has shown that periodontitis is consistently linked to adverse systemic health outcomes across cardiovascular, autoimmune, neurological, metabolic, and inflammatory domains. Evidence from cohort studies, interventional trials, genetic analyses, and microbiome research supports the interpretation that periodontal disease is a multisystem exposure characterised by persistent inflammation rather than a localised oral condition. Chronic periodontitis was associated with higher risks of hypertension and atrial fibrillation, while recovery or improvement in periodontal status was linked to partial risk reduction, suggesting that this exposure may be dynamic and potentially modifiable. Interventional findings further indicated that systemic inflammatory markers were reduced following periodontal therapy, whereas cardiometabolic changes were small or inconsistent, supporting inflammation-mediated pathways rather than direct metabolic regulation.

Periodontal disease and oral microbial dysbiosis were strongly linked to autoimmune outcomes, particularly rheumatoid arthritis, and changes in microbiome composition and autoantibody production have been observed before clinical disease onset, including in longitudinal dental intervention studies demonstrating treatment-related microbial shifts [7,8]. The findings support the interpretation that periodontal tissues may function as sites of immune activation and microbial perturbation that contribute to systemic autoimmunity, as demonstrated in longitudinal and intervention-based microbiome studies [7]. Neurological associations also suggest that chronic periodontal inflammation may contribute to cumulative vascular and inflammatory mechanisms relevant to dementia risk. Periodontitis may increase systemic inflammatory burden, disrupt immune homeostasis, and promote endothelial dysfunction, thereby increasing susceptibility to chronic systemic diseases. These relationships have important clinical and population health implications. Periodontitis emerges as a potentially modifiable risk factor for systemic diseases characterised by persistent inflammation, immune dysregulation, and vascular dysfunction [38]. The association between elevated inflammatory burden and endothelial impairment supports the need for integrated care strategies that include periodontal assessment as part of cardiovascular, metabolic, and autoimmune disease management [39]. Preventing periodontal disease early in its progression may also reduce systemic inflammatory load and related disease risk [40]. These findings reinforce oral health as an essential component of overall health rather than an isolated clinical domain.

The findings are consistent with a growing body of literature describing links between periodontal disease and systemic outcomes, including hypertension, autoimmune disease, and metabolic dysregulation [38]. Patterns of elevated inflammatory markers, increased autoimmune risk, and altered oral microbiome profiles have been reported across diverse populations and study designs [41]. The limited cardiometabolic changes observed after periodontal therapy are also consistent with prior observations that systemic benefits may be less pronounced in high-risk groups with established chronic illness [42,43]. Immunological and microbiome-based studies, including longitudinal dental intervention research, demonstrate that periodontal tissues serve as sites of immune activation and microbial disequilibrium, contributing to systemic immune responses and disease susceptibility [7,44]. The overall consistency of findings across epidemiological, interventional, genetic, and mechanistic evidence strengthens confidence in the observed associations [45]. 

Limitations and Future Recommendations

The study designs, definitions of periodontal and systemic outcomes, used diversity in interpretation of the results, preventing quantitative synthesis. Largely observational evidence limits the causal inference and residual confounding due to lifestyle choices, and comorbidity cannot be ruled out. Differences in sample size, length of follow-up and use of diagnostic codes or self-reported data could have affected results as well.

Future research should prioritise well-designed longitudinal and mechanistic studies to clarify causal pathways linking periodontitis with systemic diseases. Greater standardisation of periodontal case definitions, outcome measures, and follow-up duration is required to improve comparability across studies. Integration of multi-omics approaches, including microbiome profiling, transcriptomic analysis, and immunophenotyping, may enhance understanding of underlying biological mechanisms. Large-scale interventional trials in high-risk populations are needed to determine the systemic clinical relevance of periodontal therapy beyond oral outcomes. Interdisciplinary collaboration between dental and medical disciplines remains essential to support integrated disease prevention and comprehensive health promotion strategies. A cost-effectiveness analysis could be incorporated to aid decision-making at the policy level. Risk stratification models incorporating periodontal indicators can be developed to help identify people at risk for elevated levels of systemic disease.

## Conclusions

Periodontitis is a chronic inflammatory disease with systemic implications that extend beyond the localised pathology of the oral cavity. The evidence synthesised proves to be stable in nature, in its relationship with negative cardiovascular, metabolic, autoimmune, neurological and inflammatory events. Patients with periodontitis also have elevated blood pressure, increased systemic levels of inflammatory response, autoimmune response, and changes in oral microbiome composition relative to periodontally healthy or treated populations. Even though periodontal therapy is effective in enhancing periodontal health and lowering systemic inflammation, changes in cardiometabolic parameters do not seem to be dramatic or substantial, indicating that indirect metabolic control is largely mediated by inflammatory and vascular pathways. Additional evidence of the role of periodontal inflammation and microbiome dysbiosis in immune activation and disease susceptibility is also found in immunological and microbiome findings, even before clinical disease occurs. The mechanistic evidence points to endothelial dysfunction, inflammatory signalling, and microbial translocation as some of the principal biological pathways that connect periodontitis with pathology in the system. These data support the idea of periodontitis as a risk factor that has multisystem effects, and emphasise the need to incorporate periodontal health with overall preventive and therapeutic interventions to decrease the burden of chronic systemic pathology.
